# Knockout of Cyclophilin-D Provides Partial Amelioration of Intrinsic and Synaptic Properties Altered by Mild Traumatic Brain Injury

**DOI:** 10.3389/fnsys.2016.00063

**Published:** 2016-07-20

**Authors:** Jianli Sun, Kimberle M. Jacobs

**Affiliations:** Department of Anatomy and Neurobiology, Virginia Commonwealth UniversityRichmond, VA, USA

**Keywords:** mild traumatic brain injury, intrinsic excitability, excitatory synaptic transmission, cyclophilin-D, cyclophilin-D knockout

## Abstract

Mitochondria are central to cell survival and Ca^2+^ homeostasis due to their intracellular buffering capabilities. Mitochondrial dysfunction resulting in mitochondrial permeability transition pore (mPTP) opening has been reported after mild traumatic brain injury (mTBI). Cyclosporine A provides protection against the mPTP opening through its interaction with cyclophilin-D (CypD). A recent study has found that the extent of axonal injury after mTBI was diminished in neocortex in cyclophilin-D knockout (CypDKO) mice. Here we tested whether this CypDKO could also provide protection from the increased intrinsic and synaptic neuronal excitability previously described after mTBI in a mild central fluid percussion injury mice model. CypDKO mice were crossed with mice expressing yellow fluorescent protein (YFP) in layer V pyramidal neurons in neocortex to create CypDKO/YFP-H mice. Whole cell patch clamp recordings from axotomized (AX) and intact (IN) YFP+ layer V pyramidal neurons were made 1 and 2 days after sham or mTBI in slices from CypDKO/YFP-H mice. Both excitatory post synaptic currents (EPSCs) recorded in voltage clamp and intrinsic cellular properties, including action potential (AP), afterhyperpolarization (AHP), and depolarizing after potential (DAP) characteristics recorded in current clamp were evaluated. There was no significant difference between sham and mTBI for either spontaneous or miniature EPSC frequency, suggesting that CypDKO ameliorates excitatory synaptic abnormalities. There was a partial amelioration of intrinsic properties altered by mTBI. Alleviated were the increased slope of the AP frequency vs. injected current plot, the increased AP, AHP and DAP amplitudes. Other properties that saw a reversal that became significant in the opposite direction include the current rheobase and AP overshoot. The AP threshold remained depolarized and the input resistance remained increased in mTBI compared to sham. Additional altered properties suggest that the CypDKO likely has a direct effect on membrane properties, rather than producing a selective reduction of the effects of mTBI. These results suggest that inhibiting CypD after TBI is an effective strategy to reduce synaptic hyperexcitation, making it a continued target for potential treatment of network abnormalities.

## Introduction

Even mild forms of traumatic brain injury (mTBI) can produce significant and long lasting disruption of cognitive function. This has been shown with the Immediate Post-Concussion Assessment and Cognitive Testing battery (ImPACT) given to mTBI patients in the emergency department (Shores et al., 2008; Peterson et al., 2009; Ponsford et al., 2011). While some improvement occurs over time, this tool shows that impairment may last weeks to months after injury (Belanger et al., 2005). More than 15% have a measurable cognitive deficit at 1 year (Kashluba et al., 2008; Lee et al., 2008). Recent studies suggest that this impairment may last for many years or decades in some patients (De Beaumont et al., 2009; Monti et al., 2013; Zhang et al., 2015).

Diffuse axonal injury (DAI) has been commonly been identified as a key neuropathological feature in both TBI patients and animal models of TBI (Oppenheimer, 1968; Povlishock and Katz, 2005; Bazarian et al., 2007; Mayer et al., 2010; Browne et al., 2011; Greer et al., 2011; Orr et al., 2016). In fact, it has been suggested to be predictive of the clinical outcome assessed with the Extended Glasgow Outcome Scale (Lee et al., 2012; Bigler, 2015). The acute pathophysiology begins with the disruption of neuronal and axonal cell membranes, which initiates a pathophysiological process of abnormal intracellular function (Werner and Engelhard, 2007; Spain et al., 2010; Kramer et al., 2016). Membrane defects cause a deregulated flux of ions, including calcium (Takahashi et al., 1981; Giza and Hovda, 2014). These ionic changes result in enhanced release of excitatory neurotransmitters, particularly glutamate (Katayama et al., 1990; Zhou et al., 2013; Giza and Hovda, 2014). To restore ionic balance, membrane pump activity increases, raising glucose consumption and depleting energy stores (Yoshino et al., 1991; Giza and Hovda, 2014; Shijo et al., 2015). This will cause calcium influx to mitochondria, impairing oxidative metabolism which can also cause acidosis and edema (Giza and Hovda, 2001; Barkhoudarian et al., 2011; Blennow et al., 2012).

A number of studies have suggested that cellular metabolism reduction occurs after even mild TBI in patients (Bergsneider et al., 2000; Giza and Hovda, 2001; Praticò et al., 2002; Vagnozzi et al., 2010) as well as in animal models (Foda and Marmarou, 1994; Tavazzi et al., 2005). Experimental evidence has linked the severity of brain injury and recovery with the extent of ATP and N-acetylaspartate (NAA) decrease and recovery (Vagnozzi et al., 2005; Tavazzi et al., 2007). The posttraumatic change in cerebral metabolism relates largely to mitochondria dysfunction and calcium overload (Tavazzi et al., 2005; Vagnozzi et al., 2010).

Mitochondria can adapt and change energy production for mild cellular stress, but when extreme, the mitochondria undergo permeability transition, wherein a permeability pore (the mitochondrial permeability transition pore (mPTP), or mPTP) opens in the inner membrane, allowing in molecules less than 1500 kD (Halestrap, 2009; Brenner and Moulin, 2012). Once this occurs, ATP can become depleted and the cell can then undergo necrotic death (Halestrap, 2009). In fact, the mPTP is a key effector of cell death associated with many neurological diseases, including TBI, ischemia, stroke and neurodegenerative disorders (Griffiths and Halestrap, 1993; Lifshitz et al., 2004; Baines et al., 2005; Schinzel et al., 2005; Osman et al., 2011; Rao et al., 2014). Cyclosporine A interacts with cyclophilin-D (CypD) to block the mPTP (Nicolli et al., 1996). Blocking the mPTP with cyclosporine A or its analog NIM811 given either pre- (Büki et al., 1999; Okonkwo and Povlishock, 1999) or post- TBI has been shown to be neuroprotective in animal models of TBI (Mbye et al., 2009; Kilbaugh et al., 2011; Readnower et al., 2011). CypD induces the opening of the mitochondrial pore by sensitizing it to calcium (Bernardi and Di Lisa, 2015). Thus manipulation of CypD represents a target for controlling this pathway to energy-depletion and cell damage. This role of CypD in affecting mitochondrial permeability transition has been confirmed in studies utilizing CypD knockout mice (Baines et al., 2005; Gainutdinov et al., 2015). Studies have also shown that the absence of CypD attenuates mitochondrial and neuronal perturbation, and ameliorates learning and memory in Alzheimer’s disease (AD; Du et al., 2008). CypD inactivation also protects axonal damage in experimental autoimmune encephalomyelitis (Forte et al., 2007). A recent study noted that axonal injury was reduced after mTBI in cyclophilin-D knockout (CypDKO) mice (Hånell et al., 2015b). What has not yet been explored is whether CypDKO affects physiological neuronal function. Previously, we reported altered intrinsic and synaptic properties of layer V pyramidal neurons in somatosensory cortex 1 and 2 days after mTBI in yellow fluorescent protein (YFP)-H mice (Greer et al., 2012; Hånell et al., 2015a). This “H” mouse strain has YFP in a subset of layer V pyramidal neurons (Feng et al., 2000), allowing visualization of the full morphology, including the status of the axon in live (or fixed) tissue. By using CypD knockout mice crossed with YFP-H mice, here we evaluated whether CypD knockout ameliorates the intrinsic and synaptic abnormalities induced by mild fluid percussion injury 1 and 2 days after injury.

## Materials and Methods

### Experimental Animals

We crossed two existing mouse lines to study the effect of inhibition of the mPTP opening in neural function after mTBI. The first mouse-line expresses YFP (homozygous) under control of the Thy1-promoter (YFP-H, Jackson Labs, Bar Harbor, ME, USA). The second line consisted of CypD knock-out mice. The gene encoding CypD is ppif and thus these mice are also known as ppif^−/−^. CypD genotyping, crossing and mouse-line maintaining were managed by Dr. Povlishock’s laboratory (Hånell et al., 2015b). The two lines were crossed and followed by identification of YFP expressing *ppif^−/−^* mice. The offspring of this cross that were positive for YFP expression were used for this study and are referred to here as CypDKO/YFP-H. The age of the mice used in the study was 6–8 weeks old. The mice were grouped in 12 h/12 h non-reversed light cycle on corn cob bedding with continuous free access to food and water. All animal procedures were approved by the institutional animal care and use committee (IACUC) of Virginia Commonwealth University.

### Central Fluid Percussion Injury

Mild central fluid percussion injury was induced as described previously (Greer et al., 2011). Animals were anesthetized with 4% isoflurane in 100% O_2_. Anesthesia was maintained with 2% isoflurane during the surgery. The body temperature was maintained at 37°C by a thermostatically controlled heating pad (Harvard Apparatus, Holliston, MA, USA). Pulse rate, respiratory rate, and blood oxygenation were monitored intraoperatively via a pulse oximetry sensor (STARR Life Sciences, Oakmont, PA, USA). A 3.0 mm circular craniotomy was made along the sagittal suture midway between Bregma and lambda with dura intact (IN). This location consistently produces DAI throughout primary somatosensory cortex (see Greer et al., 2011). A sterile Leur-Loc syringe hub made from a 20 gauge needle was affixed to the craniotomy site using cyanoacrylate and dental acrylic, then filled with saline to keep dura moisture. After the dental acrylic hardened, topical bacitracin and lidocaine ointment were applied to the incision site. This surgery required 45–75 min. The animal was then removed from anesthesia and monitored in a warmed cage until fully ambulatory (60–90 min of recovery). Injury or sham procedure was applied immediately after recovery.

For the injury induction, each animal was re-anesthetized with 4% isoflurane in 100% O_2_, and the hub was attached on to a fluid percussion apparatus (Custom Design and Fabrication; Virginia Commonwealth University; Richmond, VA, USA). A mild severity injury (1.7 ± 0.06 atmospheres) was induced by a brief fluid pressure pulse upon the IN dura. The peak pressure was measured by the transducer (Tektronix 5111). After injury, the animals were visually monitored for recovery of spontaneous respiration. The duration of transient unconsciousness was determined by measuring the time of the following reflexes recovery: toe pinch, tail pinch, pinna, and righting. The injury was considered effectively mild when righting occurred in less than 6 min. For these experiments, the mean righting time was 1.2 ± 0.2 min for sham and 5.3 ± 0.2 min for injured mice. After recovery of the righting reflex, animals were placed in a warmed holding cage and monitored during recovery (typically ~60 min) before being returned to the vivarium. For sham injury, all of the above steps were followed with the exception of the release of the pendulum to induce the injury.

### Acute Slice Preparation and Patch-Clamp Recording

Mice were anesthetized with isoflurane and decapitated for quick brain removal 1 and 2 days after surgery. The brain was immediately chilled in ice-cold oxygenated sucrose-modified artificial cerebral spinal fluid (aCSF) containing: (in mM) 2.5 KCl, 10 MgSO_4_, 0.5 CaCl_2_, 1.25 NaH_2_PO_4_, 234 sucrose, 11 glucose, and 26 NaHCO_3_. Coronal slices, 300 μm thick were cut with a Leica VT 1200 slicer (Leica Microsystems, Wetzlar, Germany). The slices were incubated for 30–45 min at 34°C in an oxygenated aCSF containing: (in mM) 126 NaCl, 3 KCl, 2 MgCl_2_, 2 CaCl_2_, 1.25 NaH_2_PO_4_, 10 glucose, and 26 NaHCO_3_. Then the slices were remained at room temperature thereafter until placed in the recording chamber, which was maintained at 28 ± 0.5°C. Whole-cell patch-clamp recordings were performed under infrared Dodt contrast microscopy (Zeiss AxioExaminer); a 60× water-immersion objective was used to visually identify YFP^+^ layer V pyramidal neurons of primary somatosensory cortex (beneath the injury site) with axon descending into the white matter (IN) or ending with an axonal swelling (axotomized (AX)) deep to the surface of the slice to avoid those AX by the vibratome. We have shown previously that these morphologies are easily identified in the living slice for YFP+ layer V pyramidal neurons (Greer et al., 2012). Only YFP+ neurons were chosen for recording. All layer V YFP+ neurons in the YFP-H line are expected to be pyramidal neurons and in all cases an apical dendrite was apparent, confirming that they were pyramidal neurons. The slices were continuously perfused with aCSF solution that was saturated with 95% O_2_ and 5% CO_2_. Patch electrodes (final resistances, 2–4 MΩ) were pulled from borosilicate glass (World Precision Instruments, Sarasota, FL, USA) on a horizontal Flaming-Brown microelectrode puller (Model P-97, Sutter Instruments). The intracellular solution contained (in mM): 130 K-gluconate, 10 Hepes, 11 EGTA, 2.0 MgCl2, 2.0 CaCl2, 4 Na-ATP, and 0.2 Na-GTP. The liquid junction potential was 13.7 mV and was uncorrected. Electrode capacitance was electronically compensated. For spontaneous and miniature excitatory postsynaptic current (sEPSC and mEPSC) recording, neurons were voltage clamped at −70 mV using MultiClamp 700B (Molecular Devices, Sunnyvale, CA, USA) and digitized with a Digidata 1440A and pClamp software (Molecular Devices, at 20 kHz). Tetrodotoxin (TTX, 1 μM) was included in the aCSF for mEPSC recordings. Currents were filtered at 1 kHz. Action potentials (AP) were recorded in current-clamp mode while neurons were maintained at −60 mV, filtered at 10 kHz. To obtain intrinsic and cellular property measurements, a series of hyperpolarizing and depolarizing steps were applied in current-clamp, beginning with a −200 pA step (400 ms) and then increasing by 10 pA for a total of 70 sweeps (individual depolarizing or hyperpolarizing presentation) per cell (last sweep was 490 pA). APs were present on 16 or more of the 70 sweeps. Individual sweeps had between 1 and 30 APs. Access resistance was continuously monitored. If the series resistance increased by 20% at any time, the recording was terminated.

### Data Analysis

For these experiments in CypDKO/YFP-H mice, 1–4 neurons were recorded per slice and 4–5 slices were used from each animal. A total of nine sham injured mice (71 neurons from ~40 slices) and 10 injured mice (88 neurons from ~45 slices) were used here. EPSC data analysis was performed by MiniAnalysis (Synaptosoft). For the intrinsic properties, here we show additional measures not previously reported for the YFP-H mice. In our previous publication (Greer et al., 2012), for measures of AP amplitude, threshold, and after hyperpolarization (AHP) duration, we measured only two APs on only a single sweep in response to a mid-level depolarizing current. We have since developed an analysis program that allows us to measure all intrinsic properties shown in this report for every AP on every sweep. The AHP and ADP were also determined from all recorded APs on all sweeps. We use the term rheobase here as others have used it to indicate the lowest current level that elicits an AP. Measures shown here are the mean per cell which reflects the mean for all APs in the file. We therefore used this analysis procedure for both the original YFP-H (re-analyzed with this new program) and CypDKO/YFP-H mice reported here. To compare the current CypDKO/YFP-H data to that of YFP-H mice that we have previously reported, in Figures 2, 8 only, we normalized all data to the mean of their respective control group. For the YFP-H group, we previously showed that there was no significant difference between the results for naïve and sham animals (Greer et al., 2012). Thus the YFP-H controls contain a combination of sham and naïve data. For the CypDKO/YFP-H group, only shams were studied and thus the control groups is made up of only shams. For the EPSC data reported here, we compared the data from 1 and 2 days CypDKO/YFP-H shams and found that there was no significant difference (*t*-tests, *p* > 0.05, see Supplementary Figure 1), and thus these two control groups were combined into a single CypDKO/YFP-H sham control group. For all EPSC measures, significance was tested with a 1-way analysis of variance (ANOVA). Also for EPSC data, all cell types are included together, as we have also previously shown that cell type does not affect these measures (Hånell et al., 2015a). Since the data for intrinsic properties did vary between 1 and 2 days shams for CypDKO/YFP-H, these remain separate groups for both the direct comparison with controls (Figures 4–7) as, well as for the normalization (Figure 8). Data originally published in Greer et al. (2012) was re-analyzed and normalized for the YFP-H group shown in Figure 8 and Table 1. Data originally published in Hånell et al. (2015a) was normalized for the YFP-H group in Figure 2. For each cell type identified with the pattern of AP firing, a *z-test* was used to determine if there was a significantly different percentage present in any of the subject groups (Figure 3F). For all other measures, significance was tested using 2-way ANOVAs, with a Bonferroni *post hoc* test (SPSS software from IBM), with normality assumed. Results are reported as Mean ± SEM. In the figures with data normalized to the mean of the control group (Figures 2, 8), significance shown is for comparison between the original experimental group and controls and not tested on the normalized data. It is shown in this way simply to identify which abnormalities induced by TBI were ameliorated in the CypDKO/YFP-H mice.

**Table 1:** **Intrinsic property measurements for YFP-H group**.

Measure	Control	AX 1D	AX 2D	IN 1D	IN 2D	Group *p* value
F-I Slope (Hz/pA)	0.14 ± 0.005	0.10 ± 0.01	0.24 ± 0.11	0.14 ± 0.02	0.19 ± 0.03	**0.03**
Total adaptation	2.5 ± 0.3	2.8 ± 0.9	2.0 ± 0.4	2.1 ± 0.3	2.1 ± 0.3	0.80
Late adaptation	1.2 ± 0.1	1.0 ± 0.05	1.3 ± 0.2	1.1 ± 0.1	1.7 ± 0.7	0.49
Rin (MΩ)	83.2 ± 6.8	92.1 ± 17.7	117.0 ± 10.7	137.3 ± 21.9*	118.0 ± 7.8	**0.02**
Time to 1st AP (ms)	113.7 ± 11.0	77.8 ± 19.6	93.9 ± 29.3	91.9 ± 28.7	106.2 ± 16.0	0.76
Rheobase (pA)	114.6 ± 11.0	180.0 ± 24.0*	136.7 ± 22.3	100.0 ± 17.1	93.3 ± 9.9	**0.01**
AP threshold (mV)	−38.9 ± 0.9	−36.0 ± 1.1	−27.2 ± 2.6*	−34.5 ± 1.0	−32.7 ± 1.5*	**0.00**
AP amplitude (mV)	107.5 ± 0.8	112.1 ± 0.7	115.4 ± 1.3	108.6 ± 4.7	104.7 ± 3.7	**0.02**
AP overshoot (mV)	46.8 ± 0.8	51.2 ± 0.8	54.3 ± 0.9	47.1 ± 4.3	43.6 ± 3.6	**0.01**
AHP amplitude (mV)	13.1 ± 1.1	14.9 ± 1.5	22.5 ± 3.2	18.4 ± 1.9	20.1 ± 1.8	**0.03**
AHP time to peak (ms)	13.3 ± 0.7	12.8 ± 1.4	13.9 ± 2.5	13.7 ± 1.7	10.3 ± 1.1	0.38
AHP rise time (ms)	6.9 ± 0.4	6.0 ± 1.1	6.7 ± 1.6	6.6 ± 1.2	5.2 ± 1.0	0.63
% APs with DAPs	61 ± 5	40 ± 10	39 ± 9	37 ± 9	48 ± 11	0.07
DAP amplitude (mV)	16.5 ± 0.9	20.6 ± 2.0	23.7 ± 2.4	19.0 ± 0.9	22.3 ± 2.5	**0.03**
DAP time to peak (ms)	4.9 ± 0.4	5.5 ± 0.5	5.1 ± 0.7	6.1 ± 1.0	4.4 ± 0.7	0.50

Note that for this dataset we have shown that sham-injured did not differ from naïve and thus these two groups were combined for the single control group here. Shown are mean ± SEM. N = 30 control, 23 AX 1D, 7 AX 2D, 13 IN 1D, and 8 IN 2D. 1-way ANOVA used to assess group significance and p value shown at right. *Significantly different from control, based on Bonferroni post hoc test, p < 0.05.

## Results

### CypDKO Eliminated the Increase of Excitatory Synaptic Activity After mTBI

**Figure 1:**
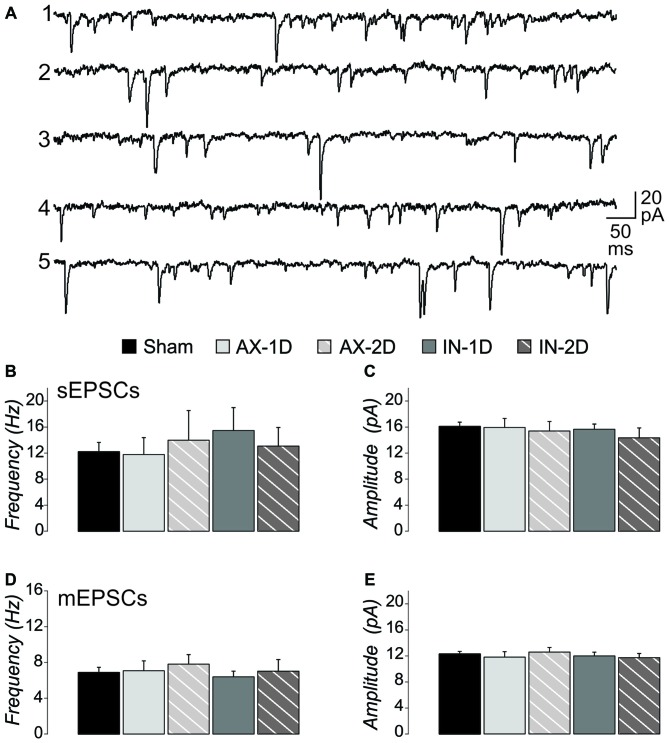
**When applied in cyclophilin-D knockout (CypDKO)/yellow fluorescent protein (YFP)-H mice, mild traumatic brain injury (mTBI) does not increase excitatory post synaptic current (EPSC) frequency. (A)** Examples of spontaneous excitatory post synaptic currents (sEPSCs) recorded from the following groups of CypDKO/YFP-H mice: (1) Sham; (2) Axotomized (AX)-ID; (3) AX-2D; (4) IN-ID; and (5) IN-2D. For this EPSC data, there was no significant difference between the ID and 2D shams and thus these were combined to form a single control sham group. The sEPSC frequency** (B)** and amplitude** (C)** were not significantly different between sham and injured groups (analysis of variance (ANOVA), *p* > 0.05, *N* = 33 sham, 9 AX-ID, 9 AX-2D, 12 IN-1D, and 8 IN-2D neurons). The miniature excitatory post synaptic current (mEPSC) frequency** (D)** and amplitude** (E)** were not significantly different between sham and injured groups (1-way ANOVA, *p* > 0.05, *N* = 32 sham, 14 AX-ID, 9 AX-2D, 12 IN-1D, and 8 IN-2D neurons).

**Figure 2:**
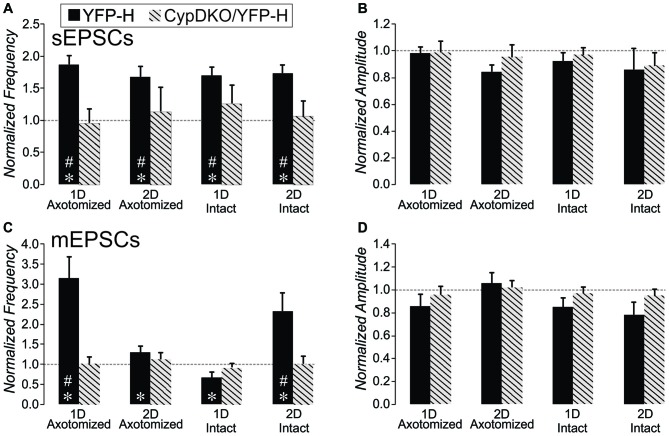
**Comparison of the effect of mTBI in YFP-H to that in CypDKO/YFP-H mice.** For each neuron from injured cortex, the values were normalized to the mean of their respective controls. For the YFP-H group, the non-normalized data was reported in Hånell et al. (2015a). For the CypDKO/YFP-H group the non-normalized data is shown in Figure 1. **(A)** The sEPSC frequency increases observed in YFP-H are ameliorated in the CypDKO/YFP-H.** (B)** The sEPSC amplitude was unaffected in either YFP-H or CypDKO/YFP-H. **(C)** The mEPSC increased frequency observed at 1 day in AX and 2 days in IN was also ameliorated in the CypDKO/YFP-H.** (D)** The mEPSC amplitude was unaffected by injury in either the YFP-H or CypDKO/YFP-H. *Significant effect of subject group (2-way ANOVA, *p* < 0.05); ^#^significantly different from control with *post hoc* analysis. Number of neurons for the CypDKO/YFP-H groups are given in the previous figure. For the YFP-H groups, for sEPSCs, *N* = 48 control, 27 AX-ID, 15 AX-2D, 16 IN-1D, and 15 IN-2D neurons, and for spontaneous mEPSCs, *N* = 15 control, 5 AX-ID, 10 AX-2D, 5 IN- 1D, and 4 IN-2D neurons.

We previously observed an increase in the frequency of excitatory synaptic currents recorded from layer V pyramidal neurons after this mild central fluid percussion injury in YFP-H mice (Hånell et al., 2015a). Here in the CypDKO/YFP-H, EPSCs appeared qualitatively similar to those in YFP-H mice (Figure 1A). In the YFP-H mice, our previous findings showed a significant increase in sEPSCs for both AX and IT neurons at both 1 and 2 days survival times. After the same injury in the CypDKO/YFP-H, this trauma-induced increase did not occur (Figure 1B). The sEPSC amplitude was also unaffected by the mTBI in CypDKO/YFP-H mice (Figure 1C). In YFP-H mice, we previously showed that the mEPSC frequency was increased at 1 day in AX and at 2 days in IN neurons (Hånell et al., 2015a). In the CypDKO/YFP-H, there was no significant difference in mEPSC frequency for any of the injured groups compared to sham (Figure 1D). The mEPSC amplitude was also unaffected by the injury (Figure 1E) in CypDKO/YFP-H mice. These results suggested that the CypDKO/YFP-H did in fact ameliorate the increased excitatory synaptic activity observed after injury in YFP-H mice. For comparison between YFP-H and CypDKO/YFP-H mice, the values for each cell in injured cortex were normalized to their respective control mean (Figure 2). In Figures 2A,C the CypDKO/YFP-H-induced amelioration of increased excitatory synaptic activity after mTBI can be seen.

### CypDKO Ameliorated Some of the Intrinsic Properties Altered After mTBI

#### Cell Types

**Figure 3:**
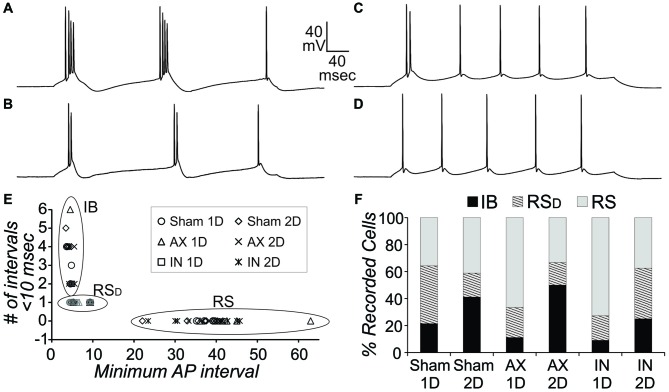
**Action potential (AP) firing pattern subtypes of YFP+ pyramidal neurons from CypDKO/YFP-H mice. (A,B)** Examples of intrinsically-bursting (IB) neurons, with high frequency bursts of APs. **(C)** Example of an regular spiking (RS_D_) neuron with doublet only at start of sweep.**(D)** Example of an RS neuron. **(E)** These three AP firing types were distinguished using the minimum AP interval and the number of AP intervals less than 10 ms. RS neurons had no AP intervals less than 15 ms. RS_D_ neurons had only one interval less than 10 ms, while IB neurons had at least two intervals of 10 ms or less.** (F)** The percent of these three types for each subject group. Percentages were not significantly different among groups (*z*-tests, *p* > 0.05). *N* = 14, 17, 9, 6, 11, and 8 neurons for sham 1D, sham 2D, AX 1D, AX 2D, IN 1D, and IN 2D, respectively.

In order to compare intrinsic properties of pyramidal neurons in different conditions, the neurons must first be divided into different subgroups based on AP firing patterns during depolarizing current steps. This is necessary since measures such as AHP amplitude and duration, rheobase, and certainly frequency per depolarization level will be consistently different depending on the firing pattern subtype. We have previously identified three subtypes for pyramidal neurons in layer V of this YFP-H tissue that were also observed here (Figure 3): (1) intrinsically-bursting (IB); (2) regular-spiking with an initial doublet (RS_D_); and (3) regular-spiking without doublet (RS). Here, firing pattern subtypes were quantitatively identified from depolarizing steps for sweeps containing 5–8 APs in the following manner. Neurons with an IB subtype had 3 or more APs per sweep with an interval of less than 10 ms (Figure 3A) or had two or more “doublets” per sweep (Figure 3B) with a doublet defined as two APs of less than 15 ms interval. The RS_D_ neurons had a single doublet per sweep (Figure 3C), while RS neurons had no APs with an interval of less than 15 ms (Figure 3D). This strategy clearly separated the three firing types (Figure 3E). All three types were observed in sham and mTBI conditions. In addition, in the CypDKO/YFP-H, the percentage of each type was similar between sham and AX as well as IN TBI groups at both 1 and 2 days survival times (Figure 3F). The previously observed loss of IB neurons for the IN 2-days after injury (IN-2D) group in YFP-H mice (Greer et al., 2012) was not present here in the CypDKO/YFP-H.

#### AP Firing Frequency Adaption

**Figure 4:**
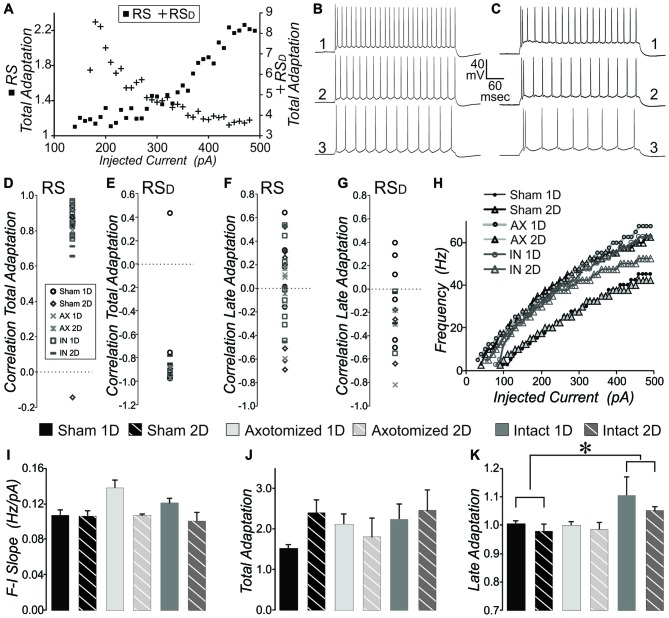
**Measures of intrinsic properties of adaptation and F-I slope in layer V pyramidal neurons from CypDKO/YFP-H mice. (A)** Plot of total adaptation vs. injected current for one example RS (squares, left axis) and one example RS_D_ (crosses, right axis) neuron, where total adaptation = frequency of first two APs in the sweep divided by the frequency of the last two APs in the sweep.** (B,C)** AP firing pattern at 450 (1), 300 (2) and 200 (3) pA for the RS **(B)** and RS_D_ **(C)** neurons plotted in **(A)**.** (D)** The correlation between total adaptation and injected current plotted for each RS neuron for all subject groups. All except one sham neuron show significantly positive correlations. **(E)** The correlation between total adaptation and injected current for all RS_D_ neurons from all groups. All except one sham neuron have a significantly negative correlation.** (F)** Correlation between late adaptation and injected current for all RS neurons. **(G)** Correlation between late adaptation and injected current for all RS_D_ neurons.** (H)** Example plots for typical individual neurons of sweep frequency (across the 400 ms depolarizing step) vs. injected current. **(I)** Mean F-I Slope for RS neurons. A 2-way ANOVA showed an effect of survival day, but no effect of subject group (significance at *p* < 0.05).** (J)** Total adaptation for RS neurons. A 2-way ANOVA showed no significant effects. **(K)** Late adaptation for combined RS and RS_D_ neurons. A 2-way ANOVA with Bonferroni *post hoc* showed a significant greater late adaptation for intact (IN) compared to sham neurons (*p* < 0.05). N for **(D,F)** = 5 Sham 1D, 7 Sham 2D, 6 AX ID, 3 AX 2D, 8 IN 1D, and 4 IN 2D neurons. N for **(E,G)** = 6 Sham 1D, 3 Sham 2D, 2 AX 1D, 2 AX 2D, 2 IN 1D, and 2 IN 2D neurons. N for **(I–K)** = 11 Sham 1D, 10 Sham 2D, 8 AX 1D, 5 AX 2D, 10 IN 1D, and 6 IN 2D neurons. *Significantly different, *p* < 0.05.

AP firing frequency adaptation is an important characteristic for each neuron’s contribution to network function, particularly in instances of heightened activity levels. The RS and RS_D_ neuron firing types could also be separated based on the change in total adaptation with level of injected current. Total adaptation was calculated as the frequency of first two APs divided by the frequency of the last two APs in the sweep. Since RS_D_ neurons fire a doublet even with low levels of depolarization, their adaptation at that level is high. The firing rate occurring after the doublet increases with greater levels of injected current (see Figure 4C) while the doublet frequency is maintained. This means that the total adaptation will be increasingly lower with greater levels of depolarizing current injection (see RS_D_ cell, crosses plotted against right axis in Figure 4A). In RS neurons in contrast, while the overall frequency increases with greater levels of depolarization, the frequency increases most for the first two APs (Figure 4B). This means that total adaptation will increase with increasing levels of depolarization (see RS, filled squares plotted against left axis in Figure 4A). Calculation of the correlation between total adaptation and depolarizing current level typically produced a positive value for RS but a negative value for RS_D_ neurons (Figures 4D,E). With the exception of a single RS and a single RS_D_ neuron, this was true for all layer V pyramidal neurons from all subject groups. This further confirms our quantitative method of separating pyramidal neuron firing types. For measures such as total adaptation that were affected by the beginning doublet, population means were then compared between subject groups only for the RS neurons (since there were a greater number of these than RS_D_). The mean total adaptation for RS neurons was not significantly different between injured and sham conditions (Figure 4J, 2-way ANOVA, no significant effects, *p* > 0.05).

Late adaptation was calculated as the frequency of the 4th and 5th AP divided by the frequency of the last two APs in sweeps with 8 or more APs. There was no consistent correlation between late adaptation and level of injected depolarizing current for any subject group whether examined in RS or RS_D_ neurons (Figures 4F,G). When the population means were examined for late adaptation, there was no significant difference between injured and controls in YFP-H mice (see Figure 8C). However, in the CypDKO/YFP-H, a 2-way ANOVA showed a significant effect of subject group and Bonferroni *post hoc* analysis showed that there was a greater adaptation for IN mTBI neurons compared to sham (Figure 4K, *p* < 0.05).

#### Slope of the Frequency vs. Injected Depolarizing Current

We previously described a significant increase in the slope of the frequency (calculated across the full 400 ms depolarization) vs. injected depolarizing current (F-I Slope) for IN-2D compared to controls in YFP-H mice (Greer et al., 2012). Here, in the CypDKO/YFP-H cortex, this effect was not observed (Figure 4I). Both RS and RS_D_ cells were included in this measure. In fact a 2-way ANOVA showed an effect of day only, with no difference between subject groups. Examples of the plot of frequency vs. injected current for individual cells from each subject group are shown in Figure 4H.

#### Basic Membrane Properties

**Figure 5:**
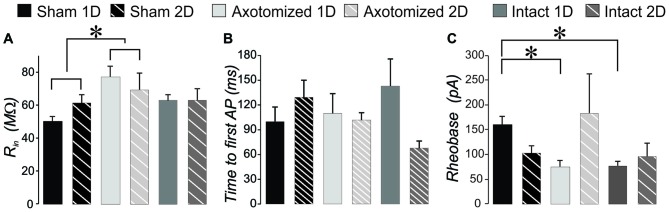
**Intrinsic properties that reflect basic neuronal membrane properties for injured compared to sham controls in CypDKO/YFP-H mice. (A)** A 2-way ANOVA showed a significant effect of subject group for input resistance (*p* < 0.02). Bonferroni *post hoc* analysis showed that the AX group had a significantly greater input resistance compared to shams. **(B)** Time to the peak of the first AP measured from beginning of depolarizing current for the rheobase sweep. A 2-way ANOVA showed no significant differences (*p* > 0.05).** (C)** Rheobase is the lowest current level to generate an AP. A 2-way ANOVA showed no effect of subject group or survival time, but there was a significant interaction of these two. *Post hoc* analyses including Bonferroni method showed that both AX and IN neurons had a significantly lower rheobase at 1 day post injury. *N* = 11 Sham ID, 10 Sham 2D, 8 AX ID, 5 AX 2D, 10 IN ID, and 6 IN 2D neurons.*Significantly different, *p* < 0.05.

Basic membrane properties of input resistance, rheobase and time to first AP at rheobase were compared between sham and injured animals for the combination of RS and RS_D_ neurons. Input resistance was significantly greater in YFP-H mice and was not corrected in the CypDKO/YFP-H cortex (see Figure 8D). In the CypDKO/YFP-H, a 2-way ANOVA with Bonferroni *post hoc* showed a significantly greater input resistance in AX neurons compared to sham (Figure 5A). The IN neurons were not significantly different from sham. Time to first AP at rheobase was variable between neurons within a group and not significantly different between subject groups in CypDKO/YFP-H (Figure 5B). In YFP-H mice rheobase was significantly increased specifically in the AX 1-day after injury (AX-1D) group (see Figure 8F). In the CypDKO/YFP-H cortex this effect was reversed, such that rheobase was significantly decreased compared to sham for both AX-1D and IN 1-day after injury (IN-1D) (Figure 5C).

#### Action Potential Properties

**Figure 6:**
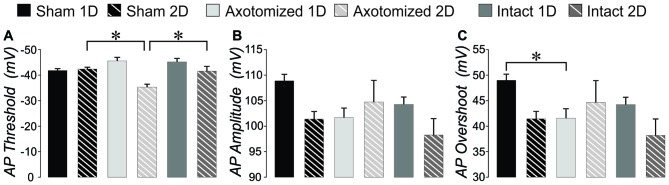
**AP measures: injured compared to shams in CypDKO/YFP-H mice. (A)** AP threshold averaged across all APs evoked per neuron. A 2-way ANOVA showed a significant effect of survival day (*p* < 0.005) and the interaction of subject group and survival day (*p* < 0.005). Bonferroni *post hoc* analyses showed that the AX-2D group had a significantly more depolarized AP threshold compared to sham-1D and IN-1D.** (B)** For the measure of AP amplitude, a 2-way ANOVA showed a significant effect for day only (*p* < 0.05). **(C)** For AP overshoot, a 2-way ANOVA showed a significant effect of day (*p* < 0.05) and a significant interaction between subject group and day (*p* < 0.05). Bonferroni *post hoc* analyses showed that AX-ID had a significantly smaller overshoot compared to sham-ID (*p* < 0.05). *N* = 11 Sham ID, 10 Sham 2D, 8 AX 1D, 5 AX 2D, 10 IN ID, and 6 IN 2D neurons. *Significantly different, *p* < 0.05.

We previously found significant differences in AP properties in YFP-H mice after injury, which included an increased AP amplitude and overshoot (Greer et al., 2012). While we found no difference in AP threshold originally, when measured for only a few APs per cell, here we re-evaluated the YFP-H mice data. When the AP threshold was measured for all APs in the file and then averaged per subject group, a 2-way ANOVA showed a significant effect of subject group and Bonferroni *post hoc* showed that AP threshold for both AX 2-day after injury (AX-2D) and IN-2D was significantly more depolarized (less than 1.0 when normalized to YFP-H controls, Figure 8G). In CypDKO/YFP-H mice this effect was ameliorated only for the IN-2D group (Figure 6A). A 2-way ANOVA showed a significant effect of day (*p* < 0.005) and a significant interaction between subject group and day (*p* < 0.005). Bonferroni *post hoc* analysis showed that AP threshold was significantly more depolarized for the AX-2D group compared to both the sham-2D and IN-2D. in the CypDKO/YFP-H mice. We previously found an increase in AP amplitude and overshoot in YFP-H injured mice. We have also since found that these effects are likely to due to an increase in the amplitude of the sodium conductance in these cells. These effects were ameliorated in the CypDKO/YFP-H mice. There was no significant difference between subject groups for AP amplitude and the increased overshoot was significantly reduced in AX-1D and not significantly different from sham in the other subject groups (Figures 6B,C, 8H,I).

#### AHP and Depolarizing After Potential (DAP)

**Figure 7:**
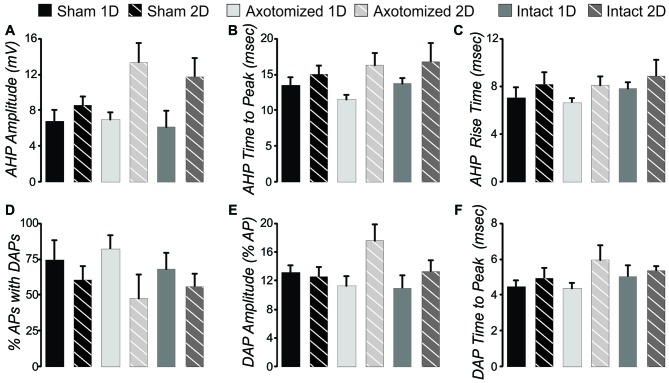
**After hyperpolarization (AHP) and depolarizing after potential (DAP) measures in the CypDKO/YFP-H. (A)** AHP amplitude calculated as the minimum voltage following the AP minus the baseline voltage prior to the depolarizing step. Thus, larger values represent more depolarized AHPs and likely lower levels of K+ current. A 2-way ANOVA showed an effect of survival day but no effect of subject group.** (B)** AHP time to peak measured relative to the AP peak. A 2-way ANOVA showed an effect of survival day but no effect of subject group (significance < 0.05). **(C)** There were no significant effects for AHP rise time (2-way ANOVA, *p* > 0.05).** (D)** The percent of APs with DAPs was calculated within individual neurons for all APs produced from that neuron. There were no significant effects for this measure (2-way ANOVA, *p* > 0.05). **(E)** DAP amplitude was calculated as a percentage of the AP amplitude. A 2-way ANOVA showed an effect of survival day but no effect of subject group (*p* < 0.05).** (F)** DAP time to peak was measured relative to the time of the preceding AP. A 2-way ANOVA showed an effect of survival day but no effect of subject group (*p* < 0.05). *N* = 11 Sham ID, 10 Sham 2D, 8 AX ID, 5 AX 2D, 10 IN 1D, and 6 IN 2D neurons.

**Figure 8:**
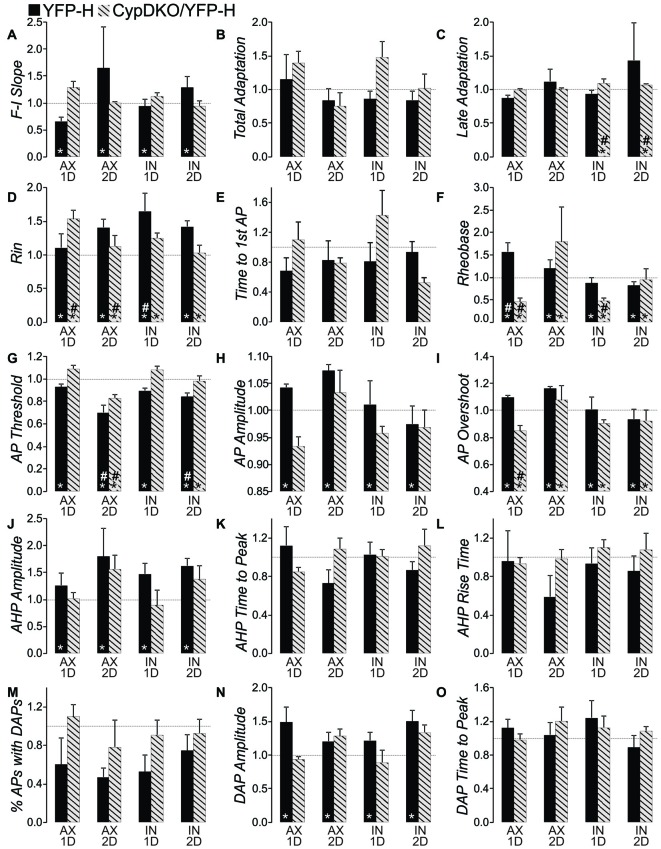
**Comparison of effects in YFP-H to those in the CypDKO/YFP-H.** **(A–O)** Measure indicated by vertical axis label. All values were normalized to the mean of their respective controls. Non-normalized data for the YFP-H group is reported in Table 1. Non-normalized data for the CypDKO/YFP-H group is shown in Figures 4–7. YFP-H shown in black bars, and CypDKO/YFP-H shown in gray bars with black stripes, with survival time and axonal status indicated on the x-axis. In all cases 2-way ANOVAs were used to compare the raw (non-normalized data) subject groups of AX and IN neurons at 1 and 2 days survival times to their respective controls. *Significant effect of subject group, ^#^significant difference of that subject group from control on Bonferroni *post hoc*. In all cases level of significance was considered *p* < 0.05. Numbers of neurons for each CypDKO/YFP-H group shown in previous figures. For YFP-H mice, *N* = 30 control, 23 AX ID, 7 AX 2D, 13 IN ID, and 8 IN 2D.

In YFP-H mice both the AHP and DAP are affected by the injury (Figures 8J,N). Note that AHP was calculated by subtracting the minimum value following the AP from the baseline period prior to the depolarizing step. Thus larger values represent ones that were more depolarized and likely reflect a smaller K+ current. For the same reason, normalized values greater than 1.0 reflect AHPs that were more depolarized than controls, again likely due to smaller K+ currents. In the CypDKO/YFP-H, 2-way ANOVAs showed effect of survival day, but no effect of subject group for either AHP amplitude or AHP time to peak (Figures 7A,B, *p* > 0.05). Thus the general effect of more depolarized AHPs found in YFP-H mice was ameliorated in the CypDKO/YFP-H. For the AHP rise time there were no significant effects in the CypDKO/YFP-H (Figure 7C). For DAPs, there were also no significant effects in the CypDKO/YFP-H for percent of APs with a DAP nor for the time to peak of the DAP measured relative to the preceding AP (Figures 7D,F). For the DAP amplitude measured as a percentage of the AP amplitude, there was a significant effect of survival day only in the CypDKO/YFP-H (Figure 7E, 2-way ANOVA, *p* < 0.05).

For a full comparison of the injury-induced effects in YFP-H vs. CypDKO/YFP-H mice, all intrinsic property data were normalized to their controls and YFP-H measures were plotted adjacent to those for the CypDKO/YFP-H (Figure 8). Indicators of significance in this figure are as measured against the raw data (shown in Table 1 for the YFP-H mice, and in Figures 4–7 for the CypDKO/YFP-H mice. This shows in the CypDKO/YFP-H complete or partial amelioration of injury-induced effects on F-I slope, input resistance, rheobase, AP threshold, amplitude and overshoot, AHP amplitude and DAP amplitude occurred. The more depolarized AP threshold for the AX-2D group failed to be ameliorated by the CypDKO/YFP-H. In addition, there was some alteration of intrinsic properties in the CypDKO/YFP-H that were not originally observed after injury in YFP-H mice. These included an increase in late adaptation, a decrease in rheobase for the IN-1D group, and a decrease in the AP overshoot for the AX-1D group.

## Discussion

Here we investigated whether CypDKO would ameliorate the intrinsic and synaptic functional alterations produced by mTBI at 1–2 day survival times. Our results suggest a partial amelioration. The increase in excitatory synaptic connections (Hånell et al., 2015a) was ameliorated, as the sEPSC and mEPSC frequencies for mTBI were similar to those for sham in CypDKO/YFP-H mice. The intrinsic properties that were ameliorated were the increased F-I slope, the increased input resistance, and the increased AP, AHP, and DAP amplitudes. Some intrinsic properties were reversed, but became significant in the opposite direction. This was true for the Rheobase and the AP overshoot. In the YFP-H mice with CypD IN, the rheobase was significantly increased only for the AX-1D group compared to controls. The increased rheobase reflects a decreased excitability, as more current is required to produce an AP. In the CypDKO/YFP-H mice, this was reversed to the point that the rheobase was significantly decreased in AX-1D compared to controls for CypDKO/YFP-H mice. There was also a significant reduction in the rheobase for the IN-1D group compared to controls for the CypDKO/YFP-H mice. These suggest an increased membrane excitability in the CypDKO/YFP-H mice selectively after mTBI. Not returned to normal levels were the AP threshold and input resistance. In addition, for the measure of late adaptation, there were significant differences with mTBI only for the CypDKO/YFP-H and not in YFP-H mice. These results suggest that the CypDKO/YFP-H does not produce a simple and complete amelioration of hyperexcitability induced by mTBI.

### Synaptic Effects

DAI is a hallmark of mTBI and is clearly observed within hours of a medial fluid percussion injury in mice (Greer et al., 2011). Sprouting of the axonal trunk of AX pyramidal neurons can be observed 1 day after the injury (Greer et al., 2011), and we have shown that this sprouting produces an increase in functional excitatory synapses in YFP-H mice (Hånell et al., 2015a). In slices from these mice, mEPSC frequency is increased after mTBI, and repeated stimulation suggests this is not due to increased probability of release (Hånell et al., 2015a). The current data suggest that in the absence of CypD, the mTBI no longer induces the increase in functional excitatory synapses (Figures 1, 2).

An effect of CypD knockout on the general function of synapses is not surprising, since CypD is concentrated at synapses and mPTP opening is more likely to occur there (Naga et al., 2007). Some work has suggested that CypD may act to sensitize the mitochondria to calcium (Gainutdinov et al., 2015). Synaptic mitochondria may also be more susceptible to calcium overload than nonsynaptic mitochrondria (Brown et al., 2006). Importantly, while mPTP opening can lead to cell death, CypD likely also acts during normal cellular processes (Barsukova et al., 2011), particularly in energy homeostasis (Elrod et al., 2010; Elrod and Molkentin, 2013; Menazza et al., 2013). Studies utilizing CypD knockout mice or reduction of CypD effectiveness with Cyclosporin A have shown altered cellular or whole animal behavioral function, although it is currently unknown whether these actions occur via CypD’s role in mPTP opening or by some other mechanism. Reducing the effectiveness of CypD with Cyclosporin A increases the resting calcium concentration, increasing normal synaptic transmission and reducing hippocampal long term plasticity levels (Levy et al., 2003). Mitochondrial uptake of calcium has also been shown to be involved in the phenomenon of post-tetanic potentiation that is due to increased transmitter release probability dependent on residual calcium in the presynaptic terminal (Dittman et al., 2000). CypD knockout also produces increased anxiety and an enhanced response in avoidance tests (Luvisetto et al., 2008). Mouri et al. (2010) have also shown that the CypDKO mice have a reduction in short term memory, using a battery of behavioral tests. After TBI, sprouting may also be considered a “normal” response to axotomy and certainly not an indicator of mPTP-induced cell death. In fact, no cell death occurs in this model of mild TBI (Singleton et al., 2002; Greer et al., 2011). The sprouting axon likely requires changes in cellular energy, such as an altered membrane potential of the mitochondria located in the growth cone (Verburg and Hollenbeck, 2008). Loss of CypD may reduce the ability of the axons to sprout.

Since CypD likely does participate in normal cellular functions, any plan for treatment must balance the potential risk of altering normal activity with preventing abnormalities after injury. Interestingly, the EPSC frequency and amplitudes were at sham levels and not below these controls, thus selectively correcting the mTBI-induced abnormality. This also occurred in the model of Alzheimer’s disease that involves overexpression of human amyloid precursor protein (Du et al., 2008). In those studies, CypDKO corrected the lack of learning in a radial water maze test. Treatment of a moderate lateral fluid percussion injury with Cyclosporine A, that effectively inhibits CypD, also improved behavioral deficits in a motor task as well as spatial learning and memory in the Morris water maze (Alessandri et al., 2002). Thus these selective corrections back to normal levels should make CypD and the mPTP a continued area of focus for potential treatments.

### Intrinsic Property Effects

In the YFP-H mice, the AX-1D mTBI group showed an increase in rheobase, suggesting a less excitable membrane. In addition, the AP threshold was more depolarized for both AX and IN groups at 2 days post-injury. The AHP amplitude was also larger for mTBI compared to controls. In contrast to this, other measures suggest increases in excitability for mTBI groups compared to controls, including the increase in input resistance, increase in AP amplitude and AP overshoot, increase in DAP amplitude and increase in F-I Slope at 2 days. The increased input resistance allows less decay of synaptic currents if the membrane capacitance is unchanged, potentially rendering the neuron more excitable (Mozzachiodi et al., 2008). Increases in DAP amplitude can enhance somatic excitability by shortening the interspike interval (ISI) and converting the somatic spike pattern into bursts terminated by high-frequency doublets (Fernandez et al., 2005). The changes in AP and AHP amplitude are likely due to altered sodium and potassium channel densities and/or current amplitudes. Other models of TBI have been shown to alter current amplitude and expression of sodium and potassium channels (D’Ambrosio et al., 1999; Hains et al., 2005; Lampert et al., 2006; Mao et al., 2010; Lei et al., 2012; Huang et al., 2013; Takahashi et al., 2013). The CypDKO reversed much of the increased membrane excitability induced by mTBI, including the AP and AHP amplitudes. Intracellular calcium levels have been shown to regulate the mRNA for certain sodium channel subunits (Vega et al., 2003) and the cell surface expression of sodium channels (Monjaraz et al., 2000). A very early response to TBI is an excessive release of glutamate (Globus et al., 1995; Koura et al., 1998), likely causing hyper-activity of NMDA receptors (Sun and Faden, 1994; Andriessen et al., 2010) and large increases in intracellular calcium (Weber, 2012) that may overwhelm the mitochondrial calcium sequestration when cyclophilin D is present. Thus the increased calcium may cause a subsequent increase in sodium channel expression. Increased sodium channel and compensatory increases in potassium channels may then directly contribute to the increased AP and AHP amplitudes. The fact that the CypDKO eliminated both the AP and AHP increased amplitude is support for the idea that the AHP increase may reflect a compensatory process. Controlling these likely effects on sodium channel expression are important, as it has been shown that blockade of sodium channels can reduce neuronal loss in models of more severe TBI (Sun and Faden, 1995).

Although the CypDKO generally caused decreases in excitability, the one exception to this was the significantly decreased rheobase at 1D for both AX and IN CypDKO/YFP-H mTBI groups compared to CypDKO/YFP-H controls (Figure 8F). Surprisingly, in these groups the AP threshold was normal relative to the CypDKO/YFP-H controls. This is a further example of the complexity of effects produced with the CypDKO. A full blockade of CypD is probably not advisable, given the effects on learning and memory (cited above) as well as the novel significant changes in late adaptation and rheobase induced by CypDKO. Nevertheless, the effectiveness of modulating the mPTP and CypD in reducing axonal injury and cell death (Matsumoto et al., 1999; Sullivan et al., 2000; Hånell et al., 2015b) as well as selectively correcting motor and learning and memory deficits suggest this type of treatment deserves continued examination. This is particularly true since, studies of TBI patients have shown that inhibiting CypD with Cyclosporine A is clinically safe and effectively increases glucose and other metabolites in extracellular fluids (Hatton et al., 2008; Mazzeo et al., 2009). In conclusion, our study suggests that in addition to the previously demonstrated structure preservation in this model (Hånell et al., 2015b), that CypDKO also provides functional protection and a substantial return to normal of many neuronal properties altered by mTBI, making continued exploration of TBI treatment via inhibition of mPTP pore opening worthwhile.

## Author Contributions

Both JS and KMJ contributed to the design of the experiments. All data from CypDKO animals was collected by JS, while control data was collected by KMJ. Both JS and KMJ contributed to data analysis and writing of the manuscript.

## Conflict of Interest Statement

The authors declare that the research was conducted in the absence of any commercial or financial relationships that could be construed as a potential conflict of interest.
