# Supplementary figures and images for: Knockout of Cyclophilin-D Provides Partial Amelioration of Intrinsic and Synaptic Properties Altered by Mild Traumatic Brain Injury
Source: Front Syst Neurosci. 2016 Jul 20;10:63. doi: 10.3389/fnsys.2016.00063 (PMC4951523; doi:10.3389/fnsys.2016.00063)

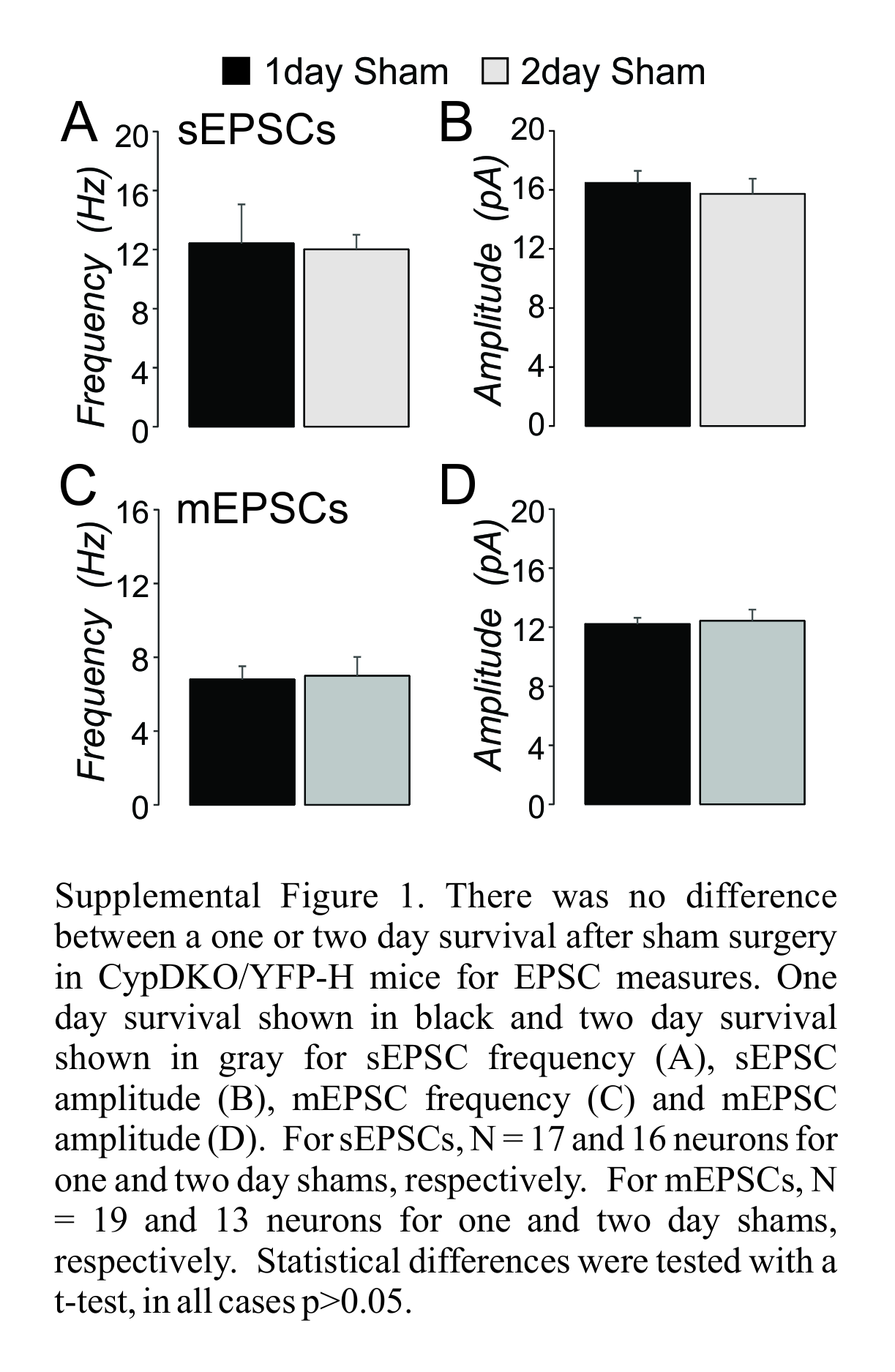

Supplement: Supplementary file 1 [file Image_1.tif]
